# Adjusting the electronic properties and contact types of graphene/F-diamane-like C_4_F_2_ van der Waals heterostructure: a first principles study†

**DOI:** 10.1039/d1ra06986a

**Published:** 2021-11-25

**Authors:** Thi-Nga Do, Son-Tung Nguyen, Cuong Q. Nguyen

**Affiliations:** Laboratory of Magnetism and Magnetic Materials, Advanced Institute of Materials Science, Ton Duc Thang University Ho Chi Minh City Vietnam dothinga@tdtu.edu.vn; Faculty of Applied Sciences, Ton Duc Thang University Ho Chi Minh City Vietnam; Faculty of Electrical Engineering Technology, Hanoi University of Industry Hanoi 100000 Vietnam; Institute of Research and Development, Duy Tan University Da Nang 550000 Vietnam nguyenquangcuong3@duytan.edu.vn; Faculty of Natural Sciences, Duy Tan University Da Nang 550000 Vietnam

## Abstract

Motivated by the successful exfoliation of two-dimensional F-diamane-like C_4_F_2_ monolayer and the superior properties of graphene-based vdW heterostructures, in this work, we perform a first principles study to investigate the atomic structure, electronic properties and contact types of the graphene/F-diamane-like C_4_F_2_ heterostructure. The graphene/C_4_F_2_ vdW heterostructure is structurally stable at room temperature. In the ground state, the graphene/C_4_F_2_ heterostructure forms n-type Schottky contact with a Schottky barrier height of 0.46/1.03 eV given by PBE/HSE06. The formation of the graphene/C_4_F_2_ heterostructure tends to decrease in the band gap of the semiconducting C_4_F_2_ layer, suggesting that such a heterostructure may have strong optical absorption. Furthermore, the electronic properties and contact types of the graphene/C_4_F_2_ heterostructure can be adjusted by applying an external electric field, which leads to the change in the Schottky barrier height and the transformation from Schottky to ohmic contact. Our findings reveal the potential of the graphene/C_4_F_2_ heterostructure as a tunable hybrid material with strong potential in electronic applications.

## Abstract 2

We perform a first principles study to investigate the atomic structure, electronic properties and contact types of the graphene/F-diamane-like C_4_F_2_ heterostructure.

## Graphical abstract

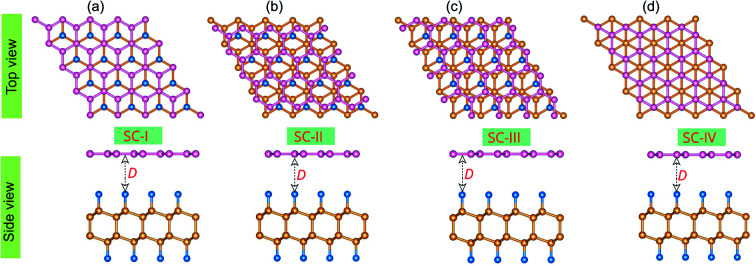

## 1 Introduction

The development of science and technology has opened the door for wide ranging materials science and engineering, especially the science of novel two-dimensional (2D) materials. Through the application of modern science and technology, such as exfoliation^1–3^ and chemical vapor deposition (CVD),^4–6^ many novel 2D materials have been successfully fabricated and investigated. Graphene, a single-atom-thick 2D material was successfully discovered by mechanical exfoliation of graphite in 2004.^7^ After such a discovery, a lot of novel 2D materials with unusual physical and chemical properties and wide range of potential applications, were discovered and studied, including transition metal dichalcogenides,^8,9^ phosphorene,^10,11^ MXenes^12,13^ and Janus-like 2D materials.^14,15^ Owing to their outstanding physical and chemical properties, 2D materials are promising candidates for electronics, optoelectronics and photocatalytics.^16–18^ However, the aforementioned 2D materials have some disadvantages that hinder their application in many advanced technologies. For instance, the lack of a band gap in graphene limits its application in high-performance nanodevices like electronics and photoelectronics.^19^ A small carrier mobility in 2D TMDs like MoS_2_ (200 cm^2^ V s^−1^ (ref. 20)) has limited its practical application. Therefore, along with finding effective strategies to modulate the properties of 2D materials, searching for novel 2D materials with the desired properties for practical applications is still challenging.

Currently, there have been many effective strategies for modulating the properties of 2D materials, such as doping,^21–24^ functionalization^25–27^ and constructing 2D van der Waals heterostructures (vdWH).^28–31^ For instance, Pierucci *et al.*^21^ demonstrated that the structural and electronic properties of the MoS_2_ monolayer can be modulated by chemical doping of hydrogen atoms, which results in the transition from an n-type to p-type semiconductor. Furthermore, Muniz *et al.* predicted that the band gap of twisted bilayer graphene can be tuned by the substitution of chemisorbed H by F atoms,^32^ by the formation of diamond superlattices^33^ and by the formation of fullerene superlattices.^34^ Sun *et al.*^35^ predicted that the ability to absorb the sunlight of 2D blue phosphorene is enhanced upon defects. Very recently, F-diamane-like C_4_F_2_ 2D materials, a new type of carbon allotrope have been successfully synthesized by a CVD method^36^ and liquid-phase exfoliation.^37^ The F-diamane-like C_4_F_2_ monolayer has been predicted to be stable under an ambient atmosphere.^36^ The F-diamane-like C_4_F_2_ possesses a semiconducting characteristic and exhibits superior carrier mobility as well as high mechanical strength and thermal conductivity.^38^ Furthermore, the electronic and transport properties of the F-diamane-like C_4_F_2_ monolayer are very sensitive to strain engineering^38,39^ and chemical functionalization.^40^ These aforementioned properties of the F-diamane-like C_4_F_2_ material make it a promising candidate for high-performance electronic and optoelectronic applications.

As we discussed above, the construction of vdW heterostructures is known to be one of the most effective tools to adjust the electronic properties of 2D materials. In particular, 2D-based vdW heterostructures can be fabricated in experiments by transfer methods^41^ or by exfoliation.^42^ A plethora of vdW heterostructures composed of two or more 2D materials have been fabricated experimentally and predicted theoretically, such as TMDs-based vdW heterostructures,^43–46^ phosphorene-based vdW heterostructures^47–49^ and graphene-based vdW heterostructures.^50–56^ Among these, graphene-based vdW heterostructures have received considerable interest owing to the existence of new properties different to the constituent materials. For instance, Aziza *et al.*^57^ showed that the Dirac cone of graphene is shifted by 100 meV toward lower binding energy upon contact between graphene and GaSe. Nguyen *et al.*^56^ predicted that the contact between graphene and a BiI_3_ monolayer gives rise to the formation of an n-type Schottky contact. To date, the interfacial characteristics and the electronic properties of the contact between graphene and an F-diamane-like C_4_F_2_ monolayer, have not yet been investigated.

Motivated by the successful exfoliation of 2D F-diamane-like C_4_F_2_ monolayers and the superior properties of graphene-based vdW heterostructures, in this work, we perform a first principles study to investigate the atomic structure, electronic properties and contact types of the graphene/F-diamane-like C_4_F_2_ (graphene/C_4_F_2_) vdW heterostructure. The graphene/C_4_F_2_ vdW heterostructure is structurally and thermodynamically stable at room temperature. The formation of graphene/C_4_F_2_ vdW heterostructure gives rise to the Schottky contact, which can be adjusted by an external electric field. Our findings reveal the potential of graphene/C_4_F_2_ heterostructure as a tunable hybrid material with strong potential in electronic applications.

## 2 Computational details

In this work, our results for the geometric optimization, electronic properties and the band alignment of the graphene/C_4_F_2_ vdWH are calculated using first-principles calculations. All calculations are carried out in the Vienna *ab initio* simulation (VASP)^58^ and Quantum Espresso ^59,60^ simulation packages. The generalized gradient approximation (GGA)^61^ by Perdew–Burke–Ernzerhof (PBE) is employed to describe the electronic exchange and correlation. The projector augmented wave (PAW) approach^62^ is chosen to treat the core and valence electrons. According to the weak vdW interactions between layered 2D materials, the DFT-D3 method of Grimme ^63^ is adopted to better describe these interactions. The underestimation of the traditional PBE method on the band gap of 2D materials leads us to use the Heyd–Scuseria–Ernzerhof (HSE06) hybrid functional to obtain a more accurate value for the band gap. The cut-off energy for the plane-wave expansion is set to be 510 eV with a 12 × 12 × 1 *k*-point mesh. To avoid interactions between periodical slabs, we set a large vacuum thickness of 30 Å along the *z* direction. The convergence of energy and force are set to be 10^−6^ eV and 10^−3^ eV Å^−1^, respectively.

## 3 Results and discussion

**Fig. 1:**
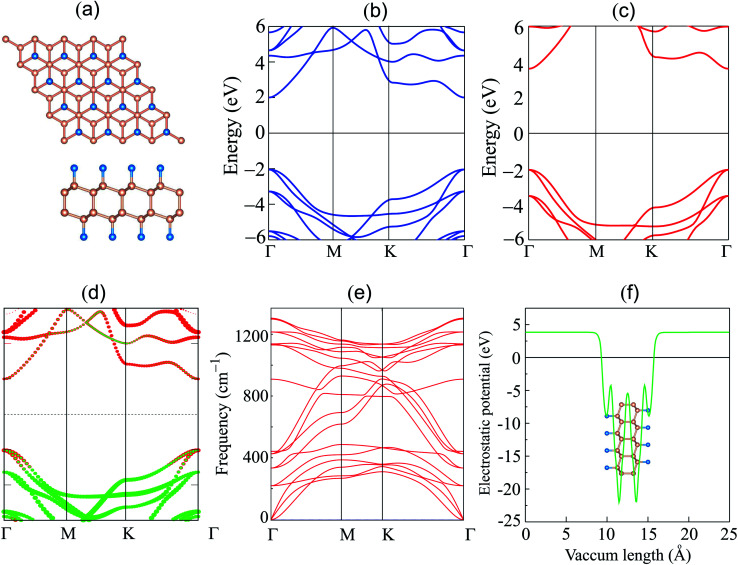
(a) Atomic structure; band structure given by (b) PBE and (c) HSE06 calculations, (d) weighted band structure; (e) phonon dispersion; and (f) electrostatic potential of the F-diamane-like C_4_F_2_ monolayer. Red and green balls in figure (d) represent the contributions of carbon and fluorine atoms, respectively.

We first explore the atomic structure and electronic properties of the F-diamane-like C_4_F_2_ monolayer. After geometric optimization, the atomic structure of single-layered F-diamane-like C_4_F_2_ is as depicted in Fig. 1(a). The F-diamane-like C_4_F_2_ monolayer has a layered buckling structure. The lattice constant of F-diamane-like C_4_F_2_ monolayer is calculated to be 2.54 Å, which shows good agreement with the experimental value^37^ and theoretical reports.^39^ The electronic band structures as well as the weighted band structure of the F-diamane-like C_4_F_2_ monolayer are depicted in Fig. 1(b–d). The F-diamane-like C_4_F_2_ monolayer exhibits a semiconducting characteristic with a direct band gap. Both the valence band maximum (VBM) and conduction band minimum (CBM) of the F-diamane-like C_4_F_2_ monolayer are located at the *Γ* point for both PBE and HSE06 method. The calculated band gap of F-diamane-like C_4_F_2_ monolayer is 4.03 eV, which shows good agreement with the previous calculations.^39,64^ The traditional PBE method is known to underestimate the band gap of 2D materials, we thus perform HSE06 calculations to obtain a more accurate band gap for the F-diamane-like C_4_F_2_ monolayer. The HSE06 band gap of the F-diamane-like C_4_F_2_ monolayer is calculated to be 5.66 eV. The weighted band structure of F-diamane-like C_4_F_2_ monolayer in Fig. 1(d) shows that both the VBM and CBM originate from hybridization between carbon and fluorine atoms. This behavior can be verified by analyzing the projected density of states (PDOS) of all atoms in the F-diamane-like C_4_F_2_ monolayer, as depicted in Fig. S1 of the ESI.† The phonon dispersion in Fig. 1(e) confirms that the F-diamane-like C_4_F_2_ monolayer is structurally stable in the ground state. The electrostatic potential of the F-diamane-like C_4_F_2_ monolayer is illustrated in Fig. 1(f). The work function of the F-diamane-like C_4_F_2_ monolayer is calculated to be 8.07 eV.

**Fig. 2:**
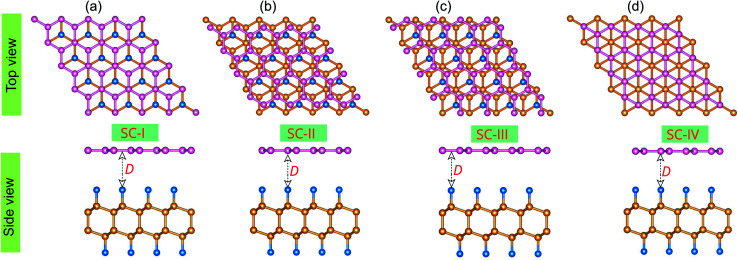
Top and side views of the optimized atomic structures of the graphene/C_4_F_2_ vdW heterostructures for different stacking configurations, (a) SC-I, (b) SC-II, (c) SC-III and (d) SC-IV. Pink, blue and orange balls represent carbon atoms in the graphene layer, fluorine and carbon atoms in the C_4_F_2_ layer, respectively. *D* stands for the equilibrium interlayer distance in the vdW heterostructure.

**Fig. 3:**
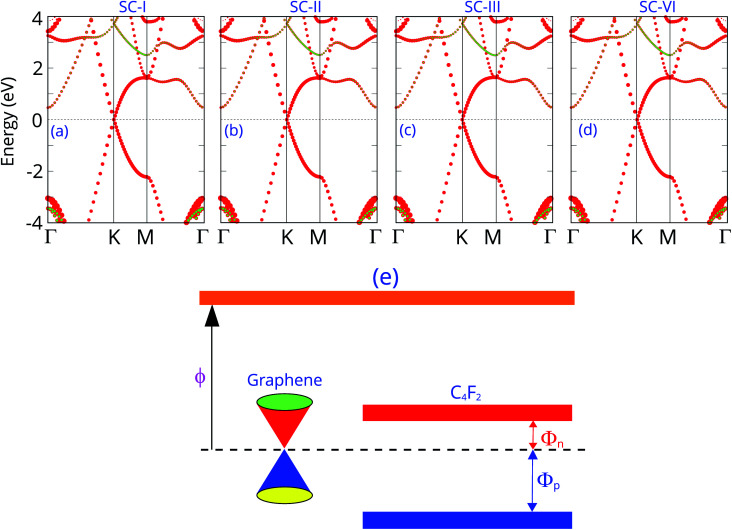
Projected band structures of the graphene/C_4_F_2_ vdW heterostructure for different stacking configurations of (a) SC-I, (b) SC-II, (c) SC-III and (d) SC-IV. Red and green balls represent the contributions of carbon and fluorine atoms, respectively. (e) Band diagram of the graphene/C_4_F_2_ vdW heterostructure.

Now, we construct the atomic structures of the graphene/C_4_F_2_ vdW heterostructure by placing graphene on top of the C_4_F_2_ layer. The same lattice parameter of graphene (2.46 Å) and C_4_F_2_ (2.54 Å) gives rise to a small lattice mismatch of about 2% in the graphene/C_4_F_2_ vdW heterostructure. The optimized atomic structure of the graphene/C_4_F_2_ vdW heterostructure for different stacking configurations, namely SC-I, SC-II, SC-III and SC-IV are depicted in Fig. 3. The equilibrium interlayer distance between graphene and the topmost layer of the C_4_F_2_ layer is defined by *D* as shown in Fig. 2. The interlayer distance for all stacking configurations is calculated to be 3.35 Å. This finding demonstrates that the structural properties of the graphene/C_4_F_2_ vdW heterostructure are insensitive to the stacking configuration. Furthermore, we find that this value of the interlayer distance is the same as that of graphite^65^ and other graphene-based vdW heterostructures.^56,66–70^ This finding suggests that the graphene/C_4_F_2_ vdW heterostructure is mainly contributed by weak vdW interactions. Such vdW interactions keep the system stable and can be obtained in future by common methods such as CVD. Furthermore, it should be noted that the weak vdW interactions between graphene and the F-diamane-like C_4_F_2_ monolayer keeps the graphene/C_4_F_2_ vdWH stable and makes the graphene surface flat. However, in reality the graphene surface may be corrugated when it is deposited on the C_4_F_2_ monolayer. The surface corrugation may affect the change in the barrier height of the contact types. However, we believe that the surface corrugation does not change the band shapes and contact types of vdWHs.

To confirm the stability of such a heterostructure, we calculate the binding energy as follows:1
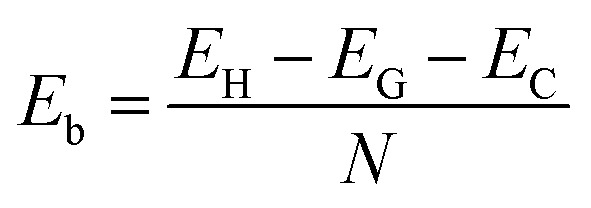
Here, *E*_H_, *E*_G_ and *E*_C_ are the total energy of the vdW heterostructure, isolated graphene and C_4_F_2_ monolayers, respectively. *N* stands for the number of carbon atoms in such vdW heterostructures. The binding energy of the graphene/C_4_F_2_ heterostructure is calculated to be −45.32 meV per C atom for SC-I, −43.96 meV per C atom for SC-II, −40.38 meV per C atom for SC-III, and −44.38 meV per C atom for SC-IV. The SC-I configuration has the lowest binding energy, indicating that it is the most energetically favorable stacking configuration. We further consider the effect of the interlayer coupling and external electric field on the electronic properties and contact types of the graphene/C_4_F_2_ vdW heterostructure for stacking SC-I configuration.

The projected band structures of the graphene/C_4_F_2_ vdW heterostructure for all stacking configurations are illustrated in Fig. 3. One can observe that the electronic band structure of the vdW heterostructure is the combination of those of the isolated constituent graphene and C_4_F_2_ monolayers. The reason of such a combination is due to the weak vdW interactions between graphene and C_4_F_2_ monolayers. Moreover, such band structures demonstrate that the stacking configurations do not affect the electronic properties of the heterostructure. Graphene maintains the metallic characteristic of a Dirac cone at the *k* point. While, the F-diamane-like C_4_F_2_ layer is the semiconductor with a direct band gap of 3.90/5.53 eV for PBE and HSE06 calculations for all stacking configurations. The contributions of each atom in the graphene/C_4_F_2_ vdW heterostructure for different stacking configurations are depicted in Fig. S2 ESI.† One can find that the Dirac cone around the Fermi level for all stacking configurations originates from the carbon atoms of the graphene layer. Interestingly, the band gap of the isolated F-diamane-like C_4_F_2_ layer is still smaller than that of the freestanding monolayer. This finding suggests that the graphene/C_4_F_2_ heterostructure may have a stronger optical absorption than that of its constituent monolayers.

More interestingly, the contact between metallic graphene and the semiconducting C_4_F_2_ monolayer gives rise to the formation of metal–semiconductor contact, which is a crucial component of high-performance electronic and optoelectronic devices. Depending on the position of the VBM and CBM of semiconducting C_4_F_2_ relative to the Fermi level of metallic graphene, the graphene/C_4_F_2_ heterostructure may form either Schottky contact or ohmic contact. The schematic diagram of the graphene/C_4_F_2_ heterostructure is illustrated in Fig. 3(e), indicating that the graphene/C_4_F_2_ heterostructure forms the Schottky contact. The Schottky contact is mainly characterized by an energy barrier, namely the Schottky barrier height (SBH). Regarding the Schottky–Mott rule,^71,72^ the n-type SBH (*Φ*_n_) and p-type (*Φ*_p_) can be calculated as: *Φ*_n_ = *E*_CBM_ − *E*_F_ and *Φ*_p_ = *E*_F_ − *E*_CBM_. The *Φ*_n_ and *Φ*_p_ are calculated to be 0.46/1.03 eV and 3.43/4.50 eV, given by PBE and HSE06 methods, respectively. This finding demonstrates that the graphene/C_4_F_2_ heterostructure exhibits an n-type Schottky contact for both PBE and HSE06 methods.

**Fig. 4:**
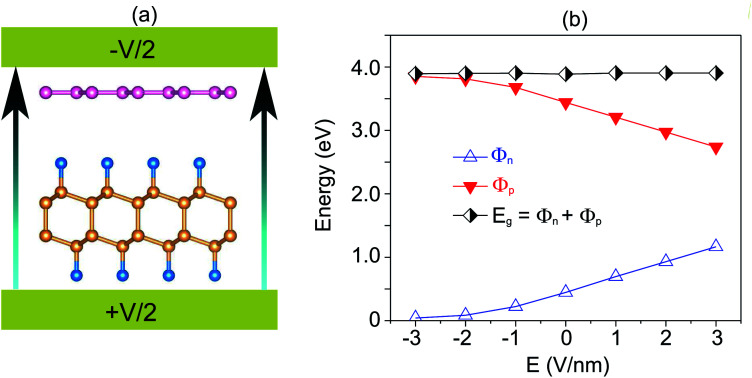
(a) Schematic diagram and (b) the variation of SBH, of the graphene/C_4_F_2_ heterostructure under an external electric field.

Furthermore, the graphene/C_4_F_2_ heterostructure is always subjected to an electric field when used as a component of electronic and optoelectronic devices. Therefore, it is important to check the effect of an external electric field on the electronic properties and contact types of the graphene/C_4_F_2_ heterostructure. The schematic model of applying an external electric field is depicted in Fig. 4(a). The electric field, pointing from the C_4_F_2_ monolayer graphene is defined as a positive direction. The change in the band gap as well as the SBH of the Schottky contact is illustrated in Fig. 4(b). One can find that in the presence of the positive electric field, ranging from 0 to +3 V nm^−1^, the *Φ*_n_ increases, while the *Φ*_p_ decreases accordingly. The change of the SBH of the graphene/C_4_F_2_ heterostructure under the positive electric field is almost linear. With the range of the positive electric field from 0 to +3 V nm^−1^, the *Φ*_n_ is still smaller than the *Φ*_p_, indicating that the n-type Schottky contact is maintained in the graphene/C_4_F_2_ heterostructure. On the other hand, when the negative electric field is applied, the *Φ*_n_ decreases, while the *Φ*_p_ increases. At the critical negative electric field of −3 V nm^−1^, the *Φ*_n_ has already fallen to nearly zero, indicating a transformation from n-type Schottky contact to n-type ohmic contact.

**Fig. 5:**
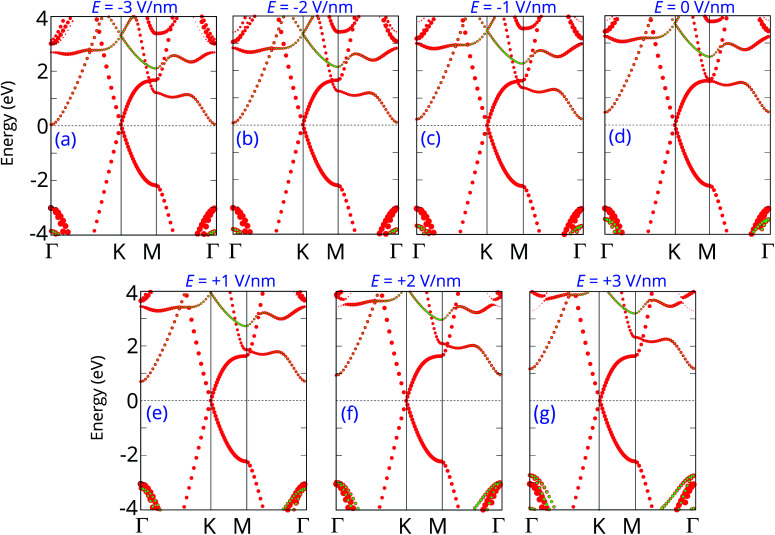
Projected band structures of graphene/C_4_F_2_ heterostructure under different electric field of (a) *E* = −3 V nm^−1^, (b) *E* = −2 V nm^−1^, (c) *E* = −1 V nm^−1^, (d) *E* = 0 V nm^−1^, (e) *E* = 1 V nm^−1^, (f) *E* = 2 V nm^−1^ and (g) *E* = 3 V nm^−1^. Red and green circles represent the contribution of C and F atoms, respectively.

The projected band structures of the graphene/C_4_F_2_ heterostructure under an electric field are plotted to provide a better understanding of the change in the electronic properties, as depicted in Fig. 5. One can see that when the positive electric field is applied, the VBM of the semiconducting C_4_F_2_ layer moves toward the Fermi level, while its CBM comes upward from the Fermi level. Such shifts cause the change in the *Φ*_n_ and *Φ*_p_ as we have discussed above. When the negative electric field is applied, the CBM is shifted downward to the Fermi level, while the VBM is moved far from the Fermi level. At the negative electric field of −3 V nm^−1^, the CBM of the semiconducting C_4_F_2_ layer crosses the Fermi level, suggesting a transformation from Schottky contact to an ohmic one. The nature of the change of band types of vdWH can be described by the change in the PFOS of all atoms, as depicted in Fig. S3 of the ESI.† All aforementioned discussions demonstrate that an external electric field gives rise not only to a change in the SBH, but also to a transition from Schottky to ohmic contact. Our findings reveal the potential of the graphene/C_4_F_2_ heterostructure as a tunable hybrid material with strong potential in electronic applications.

## 4 Conclusions

In summary, we have performed first-principles calculations to study the structural and electronic properties of the graphene/C_4_F_2_ heterostructure as well as the contact types and the effect of an external electric field. The graphene/C_4_F_2_ heterostructure is mainly characterized by weak vdW interactions with the interlayer distance of 3.35 Å and the binding energy of −45.32 meV per C atom for the most energetically favorable stacking configuration. The formation of the graphene/C_4_F_2_ heterostructure gives rise to a decrease in the band gap of semiconducting C_4_F_2_ layer and tends to the formation of the n-type Schottky contact with an SBH of 0.46/1.03 eV given by the PBE/HSE06 method. Both the contact type and SBH of the graphene/C_4_F_2_ heterostructure can be modified by applying an external electric field, which gives rise to the transformation from a Schottky contact to an ohmic one, and tends to a variation in the SBH. Our findings reveal the potential of the graphene/C_4_F_2_ heterostructure as a tunable hybrid material with strong potential in electronic applications.

## Conflicts of interest

There are no conflicts to declare.

## Supplementary Material

RA-011-D1RA06986A-s001
