# Supplementary material for: Adjusting the electronic properties and contact types of graphene/F-diamane-like C4F2 van der Waals heterostructure: a first principles study
Source: RSC Adv. 2021 Nov 25;11(60):37981–7. doi: 10.1039/d1ra06986a (PMC9044009; doi:10.1039/d1ra06986a)
Supplement: RA-011-D1RA06986A-s001 [file RA-011-D1RA06986A-s001.pdf]

Electronic Supplementary Material (ESI) for RSC Advances.
This journal is © The Royal Society of Chemistry 2021

Supplementary Information (SI)

Adjusting the electronic properties and contact types of graphene/F-diamane-like C_4F_2 van der Waals heterostructure: A first principles study

Thi-Nga Do,[1,2,*] Son-Tung Nguyen,[3] and Cuong Q. Nguyen,[4,5,*]

[1]*Laboratory of Magnetism and Magnetic Materials, Advanced Institute of Materials Science, Ton Duc Thang University, Ho Chi Minh City, Vietnam*
[2]*Faculty of Applied Sciences, Ton Duc Thang University, Ho Chi Minh City, Vietnam.*
[3]*Department of Electrical Engineering Technology, Ha Noi University of Industry, Hanoi, Vietnam.*
[4]*Institute of Research and Development, Duy Tan University, Da Nang 550000, Vietnam.*
[5]*Faculty of Natural Sciences, Duy Tan University, Da Nang 550000, Vietnam*

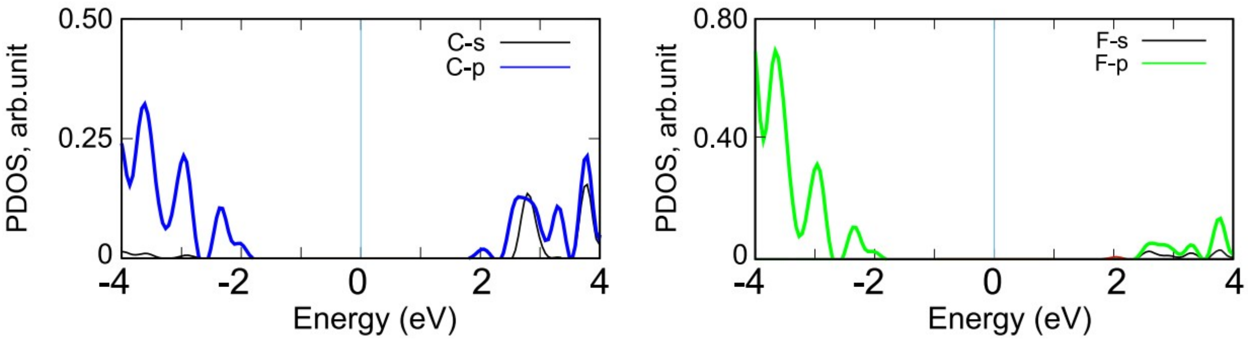

Fig. S1. Partial density of states (PDOS) of carbon and fluorine in C_4F monolayer.

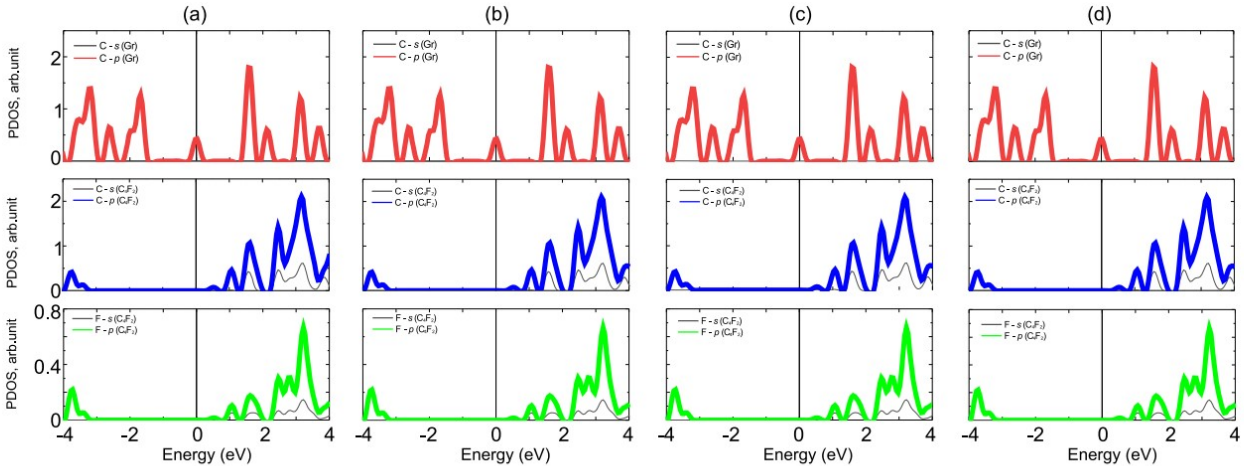

Fig. S2. Partial density of states (PDOS) of carbon in graphene layer and carbon and fluorine in C4F2 layer for different stacking configurations of (a) Stacking I, (b) Stacking II, (c) Stacking III and (d) Stacking IV. The Fermi level is set to be zero.

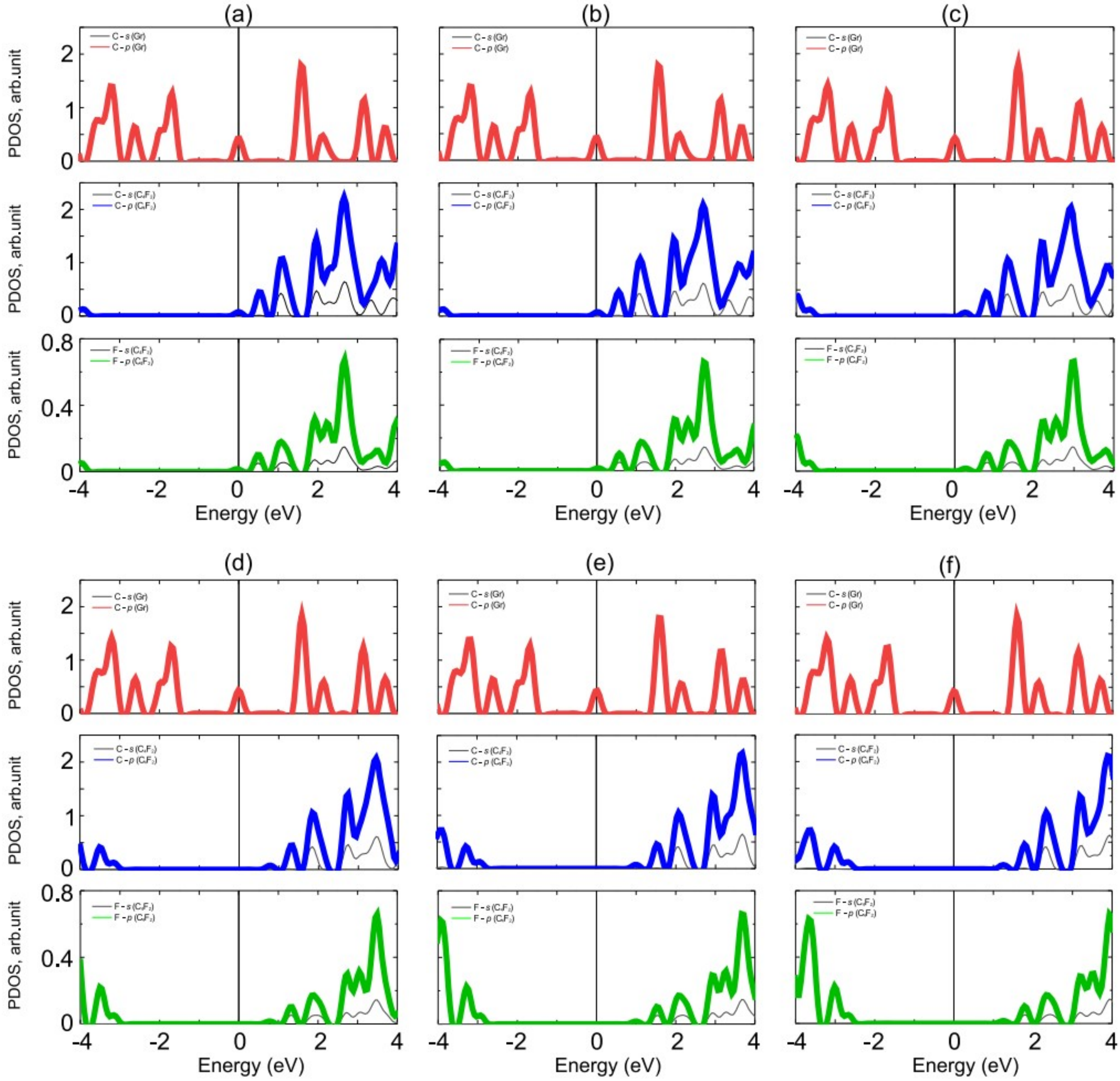

Fig. S3. Partial density of states (PDOS) of carbon in graphene layer and carbon and fluorine in C_4F_2 layer for different strength of electric field, ranging from (a) -3 V/nm, (b) -2 V/nm, (c) -1 V/nm, (d) +1 V/nm, (e) +2 V/nm and (f) +3 V/nm. The Fermi level is set to be zero.
